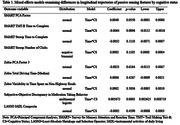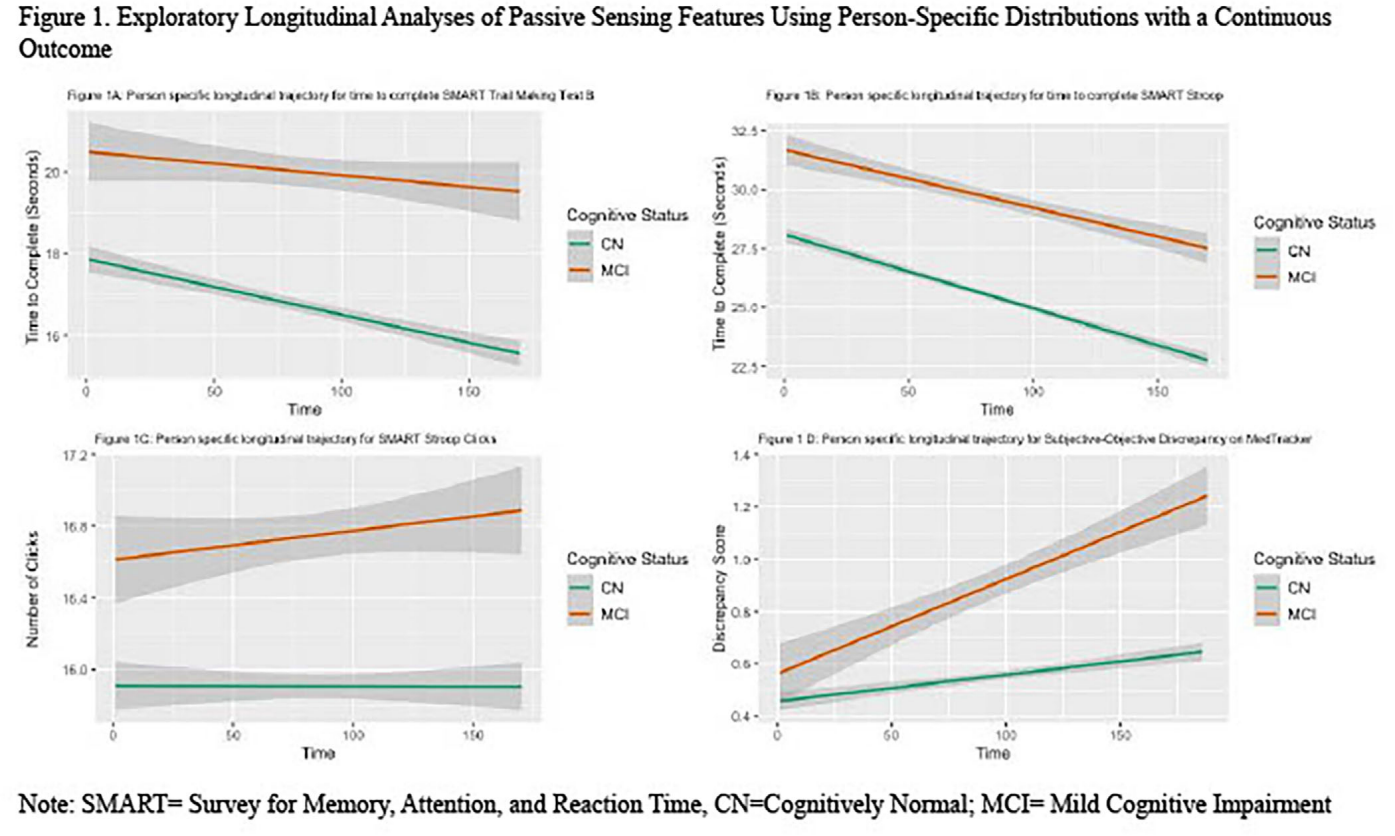# Longitudinal trajectories of passively monitored instrumental activities of daily living and high frequency digital cognitive assessment differ between cognitively normal and mild cognitively impaired older adults

**DOI:** 10.1002/alz.092318

**Published:** 2025-01-03

**Authors:** Alyssa N. De Vito, Yan Liu, Catherine H. Ju, Chao‐Yi Wu, Samuel Y. Lee, Alyssa Eichten, Anael Kuperwajs Cohen, Zachary T. Beattie, Hiroko H. Dodge, John E. Ferguson, Adriana M. Hughes

**Affiliations:** ^1^ Butler Hospital Memory and Aging Program, Providence, RI USA; ^2^ Alpert Medical School of Brown University, Providence, RI USA; ^3^ Oregon Health & Science University, Portland, OR USA; ^4^ West Virginia University, Morgantown, WV USA; ^5^ Massachusetts General Hospital, Harvard Medical School, Boston, MA USA; ^6^ University of Minnesota, Minneapolis, MN USA; ^7^ University of Minnesota School of Medicine, Minneapolis, MN USA; ^8^ Oregon Center for Aging & Technology (ORCATECH), Portland, OR USA; ^9^ Minneapolis VA Health Care System, Minneapolis, MN USA

## Abstract

**Background:**

Traditional cognitive and daily functioning measures that utilize episodic assessment schedules are less sensitive to subtle within‐person change in those at risk for Alzheimer’s disease (AD). This study aimed to evaluate whether longitudinal trajectories of high frequency cognitive assessments (HFA) and passively assessed higher order instrumental activities of daily living (IADLs) differ between those with intact cognition (CN, n = 59) and mild cognitive impairment (MCI, n = 45). An exploratory aim evaluated whether the use of person‐specific distributions would detect differences in longitudinal trajectories not captured by traditional between‐group analyses.

**Method:**

Longitudinal trajectories of six individual sensor features, three cognition/IADL domain factors, and one IADL composite measure were evaluated using mixed‐effects models with a random intercept, cognitive status (CN vs. MCI), time, and a cognitive status x time interaction as fixed effects. Person‐specific distributions were evaluated using logistic mixed‐effects models to examine the likelihood that IADL measures fell below or above person‐specific thresholds derived from the 90‐day baseline monitoring period. A change from baseline performance was indicated if a participant’s score fell below their person‐specific 10th, 20th, 30th, 40th, or 50th percentile or if a participant’s performance fell above their person‐specific mean, mode, 60th, 70th, 80th, or 90th percentile. Exploratory analyses that fit person‐specific distributions along a continuous outcome were also conducted.

**Result:**

Mixed effects models found longitudinal group differences for time to complete Trail Making Test B and the discrepancy between subjectively reported and objectively assessed pillbox use. See Table 1 for analysis results. Models that used person‐specific distributions identified differences in longitudinal trajectories for time to complete Trail Making Test B and the Stroop Test, the number of clicks on the Stroop Test, and subjective‐objective discrepancies in pillbox use between groups. See Figure 1 for exploratory analyses that used person‐specific distributions with a continuous outcome.

**Conclusion:**

HFA and passively monitored IADLs demonstrate longitudinal differences between older adults with intact cognition and those with MCI. Models that utilized person‐specific distributions identified longitudinal group differences in additional features not captured by traditional between‐group analyses. HFA approaches that use individualized distributions have potential to create more reliable and sensitive disease monitoring for AD.